# Mechanistic Analysis of the VirA Sensor Kinase in *Agrobacterium tumefaciens* Using Structural Models

**DOI:** 10.3389/fmicb.2022.898785

**Published:** 2022-05-16

**Authors:** Addison Swackhammer, Edward A. P. Provencher, Akua K. Donkor, Jessica Garofalo, Sinead Dowling, Kathleen Garchitorena, Ahkar Phyo, Nicky Ramírez Veliz, Matthew Karen, Annie Kwon, Rich Diep, Michael Norris, Martin K. Safo, B. Daniel Pierce

**Affiliations:** ^1^Department of Biology, University of Richmond, Richmond, VA, United States; ^2^Department of Biochemistry and Molecular Biology, The Pennsylvania State University, State College, PA, United States; ^3^Department of Medicinal Chemistry, School of Pharmacy, Virginia Commonwealth University, Richmond, VA, United States; ^4^Department of Chemistry, University of Richmond, Richmond, VA, United States

**Keywords:** VirA, two-component system, AlphaFold, *Agrobacterium tumefaciens*, histidine kinase

## Abstract

*Agrobacterium tumefaciens* pathogenesis of plants is initiated with signal reception and culminates with transforming the genomic DNA of its host. The histidine sensor kinase VirA receives and reacts to discrete signaling molecules for the full induction of the genes necessary for this process. Though many of the components of this process have been identified, the precise mechanism of how VirA coordinates the response to host signals, namely phenols and sugars, is unknown. Recent advances of molecular modeling have allowed us to test structure/function predictions and contextualize previous experiments with VirA. In particular, the deep mind software AlphaFold has generated a structural model for the entire protein, allowing us to construct a model that addresses the mechanism of VirA signal reception. Here, we deepen our analysis of the region of VirA that is critical for phenol reception, model and probe potential phenol-binding sites of VirA, and refine its mechanism to strengthen our understanding of *A. tumefaciens* signal perception.

## Introduction

*Agrobacterium tumefaciens*, also known as *Rhizobium radiobacter*, is a facultative pathogen that causes crown gall disease, characterized by the formation of large tumors that affect plant growth, costing millions of dollars in crop damage annually (McCullen and Binns, 2006; Nester, 2014). With its broad host range of most dicots and some monocots, *A. tumefaciens* has evolved a system to respond to various host molecules, specifically sugars and phenols excreted following a host wounding event, using a two-component system with the molecules VirA and VirG (McCullen and Binns, 2006; Hu et al., 2012; Subramoni et al., 2014). Tumor formation is caused by the transformation of the genomic DNA of a living plant cell, a process mediated through the induction of genes on the large Tumor inducing (Ti) plasmid (∼200 kb) carried by pathogenic strains of *A. tumefaciens*. A portion of this plasmid, known as transfer DNA (T-DNA), is integrated into the plant cell genome and expressed, resulting in the synthesis of opines (sugar-amino acid conjugates) that are subsequently metabolized by the pathogen.

The initiation of pathogenesis is mediated by the transmembrane histidine kinase VirA. This protein receives and transmits signals from a plant wound site as the receptor step in a typical two-component system (Duban et al., 1993; Capra and Laub, 2012). Though its structure has not been solved experimentally, modeling and genetic experiments have provided insight into its organization as a dimeric membrane-bound histidine autokinase whose architecture has been historically categorized into four domains: Periplasmic (P), Linker (L), Kinase (K), and Receiver (R) (Chang and Winans, 1992; McCullen and Binns, 2006; Lin et al., 2014). The functions of these domains have been characterized through an analysis of signal reception and the extent to which virulence is induced. Maximal signal recognition by VirA occurs in the presence of phenol derivatives, low pH, low phosphate, and simple sugars. Sugar perception at the Periplasmic region requires the presence of the periplasmic protein ChvE, a sugar-binding protein that is necessary for sensitizing VirA to incoming sugar signaling (He et al., 2009). The Linker region is necessary for reception of phenols, and successful signaling leads to auto-phosphorylation at the Kinase domain followed by activation of the downstream element, VirG.

Previous work has shown that a locus within VirA is of particular importance for the interpretation of both sugar and phenol signaling (Fang et al., 2015). In wild-type VirA, phenol signaling is necessary for the initiation of virulence and is greatly amplified by the presence of sugars. The Signal Integration Node (SIN) is a region between the Periplasmic and Linker domains of VirA that is crucial for phenol specificity (Fang et al., 2015). N-terminal truncations of the SIN result in a specificity switch between the phenols acetosyringone (AS) and dimethoxyphenol (DMP). Screens inducing random mutations to the VirA SIN have revealed several residues of importance for phenol specificity, including a tyrosine residue (Y293), which when mutated to phenylalanine, results in a switch from a Boolean AND gate (requiring sugar and phenol for response) to an OR gate (responding to either sugar or phenol) (Fang et al., 2015). This mutation, Y293F, has been found in substrains of *A. tumefaciens* that correlate with a limited-host-range and provides an elevated response to sugars and phenols (Liu, 2012; Fang et al., 2015). Further work is necessary to determine a causal link between host range, *virA* alleles, and AND/OR gating as determined through *vir* induction in the presence of inducing molecules.

While the Periplasmic region of VirA interacts with sugar-bound ChvE, phenol signals are received through the VirA Linker region. The Linker region is homologous to GAF (cGMP-specific phosphodiesterases, adenylyl cyclases, and FhlA) domains, known to bind small molecules (Ho et al., 2000; Martinez et al., 2002; Lin et al., 2014), and potentially contains binding regions for phenols (Hess et al., 1991; Duban et al., 1993; Gao and Lynn, 2007). The VirA construct without the Linker region cannot respond to phenols, though there is currently no direct biophysical evidence for this interaction (Lee et al., 1996), and additional phenol-binding proteins may play a role (Lee et al., 1992; Dyé and Delmotte, 1997; Campbell et al., 2000; Joubert et al., 2002). This remains an outstanding question in the field. Previous models of the VirA Linker have been limited by prediction software that uses only known structures to make these predictions. For instance, models using Phyre2 software show VirA homology with various GAF domains, but do not include the SIN in the prediction, as this is outside the homologous region to GAF domains (Lin et al., 2014). Recent advances made by the DeepMind Artificial Intelligence (AI) algorithm AlphaFold have confirmed the prediction that the VirA Linker is a GAF domain, while creating a comprehensive model for VirA structure which could lead toward a novel understanding of its signal coordination (Jumper et al., 2021; Varadi et al., 2021).

In this study, we use the recent AlphaFold predictions of VirA structure to contextualize our understanding of the SIN through molecular modeling and analysis of directed mutations of VirA. The model we report here has allowed us to deepen our insight into the structural architecture of how VirA governs its signal response. Additionally, we provide novel evidence to understand Boolean logic gates in VirA, including characterizing the role of pH and inhibitors in affecting virulence initiation and examining how VirA may interact with phenols. Through models of how the SIN integrates xenognostic signals from plants, we contribute to answering the outstanding questions of how VirA coordinates the virulence response.

## Materials and Methods

### Molecular Modeling

Homology molecular models of VirA were either downloaded via AlphaFold (Jumper et al., 2021; Varadi et al., 2021) or generated via Phyre2 as described previously (Kelley and Sternberg, 2009; Lin et al., 2014), and manipulated using PyMoL (The PyMoL Molecular Graphics System, version 1.7.4, Schrödinger, LLC). The Linker regions of the two models showed several pockets that we surmise could potentially bind phenol, and were therefore targeted for docking the following phenols, AS and DIMBOA using the GOLD docking program (Genetic Optimization for Ligand Docking, Cambridge Crystallographic Data Centre-CCDC, Cambridge, United Kingdom). The homology models were first energy minimized with SYBYL-X 2.1.1 (Certara USA, Inc., Princeton, NJ, United States) using the Tripos Force Field (TFF) with Gasteiger–Hückel charges and distance-dependent dielectric constant of 4.0 D/Å to an energy gradient cutoff of 0.05 kcal (mol × Å)^–1^ or 10,000 iterations. The binding pockets were defined to include all atoms within a 15 Å radius of a selected amino acid residue, and 10 solutions per ligand were generated, with no early termination nor constraints in order to obtain multiple poses within the binding site. The binding pose with the best GOLD score was selected.

### Vector Design

Parent plasmid pJZ6 was used to create pDP106, full length *virA* under the control of the P_*N*25_ promoter. Using the primers DP204 and DP164 (Supplementary Table 1) and the template pVRA8 containing *virA* (Lee et al., 1992), PCR was conducted using high-fidelity polymerase (PfuUltra II from NEB). The PCR product was digested with *Bam*HI and *Acc*65I, gel purified using the Qiagen Gel Extraction kit, and ligated into pJZ6 (Lin et al., 2014). The sequence was confirmed using eurofins genomics.

### SLIM Primer Design

Primers were designed to amplify the ∼10 kb pDP106 to introduce mutations in *virA* as indicated (Table 1 and Supplementary Table 1). These mutations were introduced into the 5′ overhang of the primers used in PCR amplification of pDP106. Four required primers were designed for each set of mutations (see Supplementary Table 1) as described previously, with two primers containing 5′ overhangs with mutation and two without (Chiu et al., 2004).

**TABLE 1:** Bacterial strains and plasmids used in this study.

Strains/plasmids	Relevant characteristics	References
***E. coli* strains**		
DH5-αβ	*recA1*, *endA1*, *lacZΔM15*	Invitrogen, Taylor et al., 1993
***A. tumefaciens* strains**		
A136	Strain C58 cured of pTi plasmid	Watson et al., 1975
A348	A136 containing pTiA6NC	Garfinkel and Nester, 1980
YHL310	A348 with *virA(Y293F)*	This study
**Plasmids**		
pVRA8	*virA* from pTiA6 in pUCD2, pBR322*ori*, IncW, Ap*^r^*	Lee et al., 1992
pRG109	*P_*N25*_-His_6_-virG*, *P_*virB*_-lacZ* in pMON596, IncP, Spec*^r^*	Gao and Lynn, 2005
pJZ6	IncW/ColE expression vector with *P*_*N25*_, Ap*^r^*	Lin et al., 2014
pDP106	*virA* in pJZ6, Ap*^r^*	This study
pDP118	*virA(Y293F)* in pJZ6, Ap*^r^*	This study
pDP143	*virA(W355A)* in pJZ6, Ap*^r^*	This study
pDP149	*virA(Y293A)* in pJZ6, Ap*^r^*	This study
pDP150	*virA(Y293G)* in pJZ6, Ap*^r^*	This study
pDP152	*virA(280AAA281, Insertion of 3 Alanine after aa 280)* in pJZ6, Ap*^r^*	This study
pDP154	*virA(Y293P)* in pJZ6, Ap*^r^*	This study
pDP159	*virA(285AAA286, Insertion of 3 Alanine after aa 285)* in pJZ6, Ap*^r^*	This study
pDP160	*virA(293AAA294, Insertion of 3 Alanine after aa 293)* in pJZ6, Ap*^r^*	This study
pDP166	*virA(W355F)* in pJZ6, Ap*^r^*	This study

### SLIM PCR Amplification

The previously described SLIM amplification was carried out with a slight modification, where two PCR reactions were performed for each mutation set. One contained the forward tailed (F_*T*_) and reverse short (R_*S*_) primers, and the other contained the forward short (F_*S*_) and reverse tailed (R_*T*_) primers. This modification eliminates the amplification of non-functional products by ensuring that no products are amplified with both tailed primers or both short primers, increasing efficiency of the amplification as well as yield. Each reaction contained 5 μL of 5× Q5 Reaction Buffer, 5 μL of 5× Q5 High GC Enhancer, 200 μM each dNTP, 0.5 μM each primer as described above, 0.5 U Q5 HF DNA Polymerase, 0.5 μL of template DNA collected via spin miniprep, and PCR-grade water up to a final volume of 25 μL. The reactions were run in a Bio-Rad C1000 Touch thermal cycler at 98°C for 30 s followed by 26 cycles of 95°C for 15 s, 57°C for 20 s, and 72°C for 5.5 min. A final extension was performed at 72°C for 10 min.

### SLIM Hybridization

The PCR products were diluted with 5 μL Buffer D (20 mM MgCl_2_, 20 mM Tris pH 8.0, and 5 mM DTT) and 10 U Dpn1. The mixtures were incubated at 37°C for 1 h. Q5 reaction buffer is known to reduce the activity of Dpn1 (NEB), so samples were digested for a longer period of time to ensure maximum cleavage possible. Q5 was still used due to the template size and need for high-fidelity sequence amplification. After digesting with Dpn1, 10 μL of each PCR reaction were combined with 10 μL of Buffer H (300 mM NaCl, 50 mM Tris pH 9.0, and 20 mM EDTA pH 8.0) and diluted with 20 μL of water. This mixture was then incubated in a Bio-Rad C1000 Touch thermocycler at 99°C for 3 min followed by 3 cycles of 65°C for 5 min and 30°C for 40 min. Immediately following this incubation, 10 μL of this reaction was transformed into NEB DH5α competent *E. coli*. To ensure the resulting colonies contained the mutant plasmid, 2 μL of each original PCR was also transformed as described above. This ensures that the Dpn1 digest functioned to remove any remaining parental DNA left in our samples. Each mutant was confirmed by sequencing.

### *Agrobacterium tumefaciens* Transformation

*Agrobacterium tumefaciens* strain A136, C58 cured of its pTi plasmid (Watson et al., 1975), has proven to be an effective background for examining how inducing signals affect the two-component system composed of VirA, VirG, and *P_*virB*_-lacZ* (Fang et al., 2015). Electrocompetent cells of A136 in 50 μL aliquots were combined with pJZ6 containing mutant *virA* and pRG109 to a final concentration of approximately 0.05 μg/μL. Afterward, they were incubated on ice for 5–10 min and then transferred into an electroporation cuvette. The samples were electroporated at 1.8 volts three times, attaining a time constant of 4.5–5.5 msec each time. Following the electroporation, 1 mL of LB without antibiotics was added to the cuvette and mixed. The culture was transferred to test tubes and incubated for approximately 2 h at 28°C with shaking. Once the incubation period was finished, 100 μL of the culture was plated on LB plates containing carbenicillin and spectinomycin. After 2–3 days, colonies appeared and were restreaked on fresh plates.

### β-Galactosidase Assays

Strains were inoculated into 4 mL LB liquid and incubated overnight at 28°C with shaking. Cultures were back-diluted to an OD_600_ of 0.1 in 2 mL of Induction Media (AB Medium pH5.5, 0.04 × AB Buffer, 1 × AB Salts) (Chilton et al., 1974; Winans et al., 1988). Inducers, phenols and/or sugars, were added as appropriate (phenols dissolved in DMSO), and cultures were incubated at 28°C with shaking for 16 h, as in previous assays (Lin et al., 2014; Fang et al., 2015). After incubation, 200 μL induction culture and 800 μL Z-buffer (0.06 M Na_2_HPO_4_ • 7H_2_O, 0.04 M NaH_2_PO_4_ • H_2_O, 0.01 M KCl, 0.001 M MGSO_4_ • 7H_2_O, 0.2% β-mercaptoethanol) were combined in a 1.7 mL tube in triplicate. To each tube, 40 μL 0.05% SDS and 40 μL chloroform was added, and tubes were mixed by inverting. Tubes were incubated at room temperature for 10 min before 200 μL o-NPG (4 mg/mL) was added to each tube. Tubes were allowed to incubate at room temperature for a sufficient amount of time to observe color change. To terminate the reaction, 200 μL 2.5 M Na_2_CO_3_ was added to each tube. Samples were then centrifuged at 13,500 rpm for 3 min to pellet cell debris, and A_420_ of the supernatant was measured. To calculate Miller Units of Activity, the following equation was used:


MillerUnits=(A•42010•37.5)/(Xmin•A)600.

### ASBr Synthesis

Acetosyringone (1.960 g, 10 mmol) was added to a three-necked round-bottom flask and dissolved in acetic acid (30 mL). The reaction was placed under N_2_ and bromine (0.462 mL, 0.927 mmol) was added to the flask. The reaction was stirred for 2 h at room temperature. After the addition of 50 mL H_2_O, the solution was extracted with 3 • 50 mL portions of ethyl acetate. The ethyl acetate was removed on a rotary evaporator. The resulting brown residue was then dissolved in a minimum amount of diethyl either and hexanes were slowly added until the mixture became turbid. The mixture was then stored at −20°C overnight and a beige precipitate formed which was filtered and collected. The remaining solid was analyzed through ^1^H NMR to confirm the presence of ASBr.

## Results

### AlphaFold Prediction of VirA Structure

**FIGURE 1:**
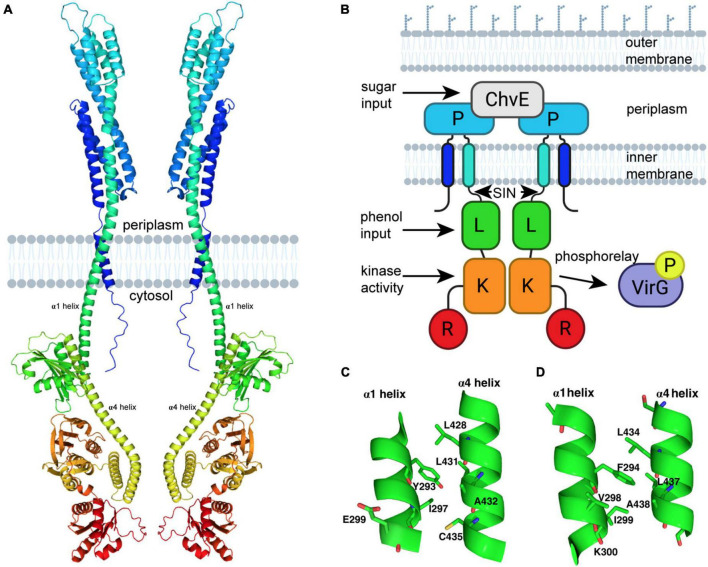
VirA modeling using AlphaFold. **(A)** Prediction of VirA (UniProt P07167) structure using AlphaFold (Jumper et al., 2021; Varadi et al., 2021). PyMoL-generated model is from the N-terminus (blue) to the C-terminus (red), and shown as a dimer with two membrane-spanning regions per VirA monomer, consistent with previous VirA studies. **(B)** Schematic model of VirA using a similar color scheme and designating phenol and sugar inputs that allow for auto-phosphorylation and two-component signaling to VirG. **(C)** VirA(Y293) position prediction using AlphaFold for wide-host-range Agrobacterium (UniProt P07167) and **(D)** VirA(F294) position prediction using AlphaFold for limited-host-range Agrobacterium (UniProt P07168), with α1 and α4 shown for each. The software program www.biorender.com was used to create a portion of these images.

The recent attempt to develop protein structural models using the DeepMind AI program AlphaFold has generated multiple predictive models for proteins that do not have a previously experimentally solved structure (Jumper et al., 2021; Varadi et al., 2021). This program has generated four models of VirA from unique *A. tumefaciens* substrains, observable on the AlphaFold repository. We present a modified version of one of these models in Figure 1A. This model was generated from VirA sequence from *A. tumefaciens* strain A348, and structural predictions of the Linker region of this protein sequence have been described previously (UniProt P07167) (Gao and Lynn, 2007; Lin et al., 2014; Fang et al., 2015). While the AlphaFold prediction algorithm shows the protein in its monomeric form, we have used previous crosslinking experiments, genetic data, and predicted membrane-spanning regions (Pan et al., 1993; Brencic et al., 2004; Nair et al., 2011) to build on the AlphaFold prediction and show VirA as a dimer imbedded in the bacterial inner membrane (see Figure 1A).

Comparing the AlphaFold homology model to the VirA architecture that has been reported previously (Figure 1B) can allow us to contextualize previous observations and experiments that have sought to define VirA function. The AlphaFold model confirms and extends previous predictions of VirA, particularly of the α-helix that encompasses the second transmembrane region (the traditional nomenclature of α1 and α4 referring to these Linker helices is kept here) (Gao and Lynn, 2007; Lin et al., 2014). AlphaFold predicts and models α1 as a helix 70 amino acids long (aa 233–302), similar to previous helix length predictions (Nair et al., 2011) – however, this model provides the first structural prediction of a mechanism to connect the periplasmic region to the Linker domain. Structural prediction programs such as Phyre2 have predicted that the Linker region is a GAF domain (Kelley and Sternberg, 2009; Lin et al., 2014; Fang et al., 2015), and the AlphaFold model builds and refines this prediction. Although the secondary structure predictions by Phyre2 and AlphaFold of the Linker models are similar, including four α-helices and several β-sheets, the SIN (aa 280–294) region was missing in the Phyre2 prediction because it occurred prior to the GAF homology region (Lin et al., 2014). The SIN region has previously been reported to be involved in the logic gating of inducing molecules (Fang et al., 2015).

Using the AlphaFold model, we can develop further insight into the SIN, and particularly the importance of amino acid 293. Most wide-host-range strains of *A. tumefaciens* have a tyrosine at position 293. The AlphaFold model shows that Y293 is located on the α1 helix and should be capable of forming extensive hydrophobic interactions with L428, L431, and C435 and a hydrophilic interaction with the amide oxygen of A432 of the opposite helix (Figure 1C). It is likely that perturbation of the Y293 position that disrupts any or a subset of these interactions could alter the relative positions of these two helices and thus affect the Linker structure, resulting in a change of phenol reception. Replacing the tyrosine with phenylalanine (which cannot participate in hydrogen-bond interaction) experimentally results in the decoupling of the sugar and phenol signals and an overall increase in VirA signal response (Fang et al., 2015). The corresponding aromatic amino acid found in limited-host-range *A. tumefaciens* strains is F294 (UniProt P07167) (Figure 1D). Indeed, our AlphaFold model suggests a slight shift in the relative positions of the two helices likely due to the absence of the hydroxyl group, which precludes this residue from forming hydrogen-bond interaction with the amide oxygen of A432. The presence of the hydroxyl group appears to play a significant role in the function of the Linker region, possibly through differences in how wide-host-range Y293 and limited-host-range F294 can interact with neighboring amino acids. Overall, there is significant homology between the sequences of limited-host-range VirA and the wide-host range VirA (Supplementary Figure 1), and AlphaFold predicts a positioning of the Linker region that is slightly different in the context of the full protein (Supplementary Figure 2). Further structural work will be necessary to interrogate these differences experimentally.

### The VirA Signal Integration Node Is Critical for Phenol Perception

**FIGURE 2:**
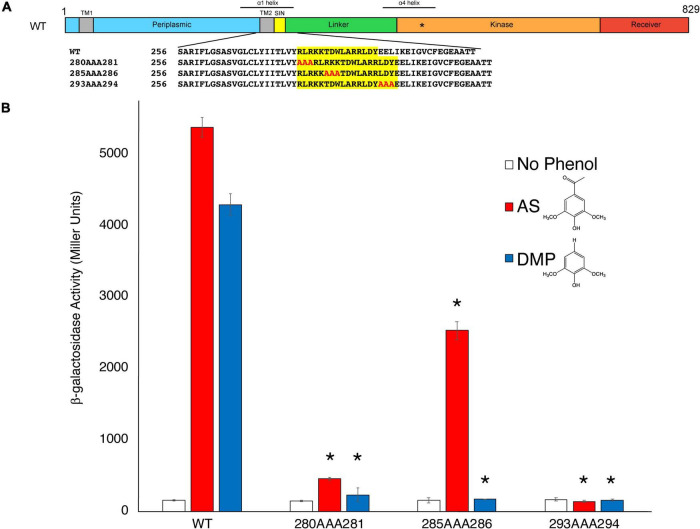
Insertions to the VirA SIN disrupt phenol perception. **(A)** A schematic of the VirA molecule (UniProt P07168), including the Periplasmic, Linker, Kinase, and Receiver domains. The Signal Integration Node (SIN), TM1/2, helices α1 and α4, and the histidine that is the site of autophosphorylation (*) are also shown. The triple-alanine insertion constructs were designed and created using SLIM (Chiu et al., 2004). **(B)** pJZ6 containing VirA*^wt^*, VirA^280AAA281^, VirA^285AAA286^, and VirA^293AAA294^ were transformed with pRG109 into *A. tumefaciens* strain A136. Strains were induced in the presence of 1% glucose, with 300 μM of the phenol acetosyringone (AS) (red bars), dimethoxyphenol (DMP) (blue bars), or DMSO (white bars), and the β-galactosidase activity was determined. Error bars represent the standard deviation of three isolates and the asterisks (*) represents a significant difference (*P* value < 0.05) when compared with the corresponding wild type value.

As discussed previously, the amino acids 280–295 of VirA, designated its Signal Integration Node (SIN), affect its coordination of sugar and phenols (Fang et al., 2015). While N-terminal deletion of the first 280 amino acids in VirA has only slight effects on its phenol response, deletion of the subsequent SIN region abrogates acetosyringone (AS) response (Fang et al., 2015). However, a VirA truncation without the first 295 amino acids (aa 295–811) is still able to respond to the phenol dimethoxyphenol (DMP) (Fang et al., 2015). Figure 2A shows a schematic of VirA, including the four regions described previously. Of note with the AlphaFold prediction is the increased length of the α1 helix, as shown above the VirA schematic (Figure 2A), relative to previous models using alternative predictive software (Lin et al., 2014; Fang et al., 2015).

To further test the importance of the SIN, and to observe how lengthening the VirA molecule in this region affects phenol response, we created mutant VirA alleles with the addition of three alanine residues after aa280 (VirA^280*AAA*281^), aa285 (VirA^285*AAA*286^), and aa293 (VirA^293*AAA*294^) using the SLIM technique (Chiu et al., 2004; Figure 2A). These mutants were designed to extend the distance between the Periplasmic and Linker regions while not adding charged residues or, hopefully, affecting secondary structure. Given that VirA lacking the 280 amino acid Periplasmic domain, the previously characterized VirA(LKR) mutant, is able to respond to AS signaling (Gao and Lynn, 2005), we hypothesized that an addition of amino acids between 280 and 281 might not affect AS signaling. This was not our finding, as our VirA^280*AAA*281^ and VirA^293*AAA*294^ mutants had their phenol-sensing activity abolished (Figure 2B). Interestingly, we found that VirA^285*AAA*286^ retained AS activity, possibly by reorienting this region in an acceptable position for signal reception. However, DMP activity was diminished in all three mutants, whereas it was previously observed that N-terminal truncations into the VirA SIN abrogated AS activity but enhanced DMP activity (Fang et al., 2015). While this further implicates this region as critically important for VirA phenol-sensing specificity, the mechanism for phenol-Linker interaction is still unclear. Additionally, it should be noted that these mutations, presumably except for VirA^285*AAA*286^, may have significant structural issues that contribute to their inability to sense phenol.

### Intermediate Logic Gating

**FIGURE 3:**
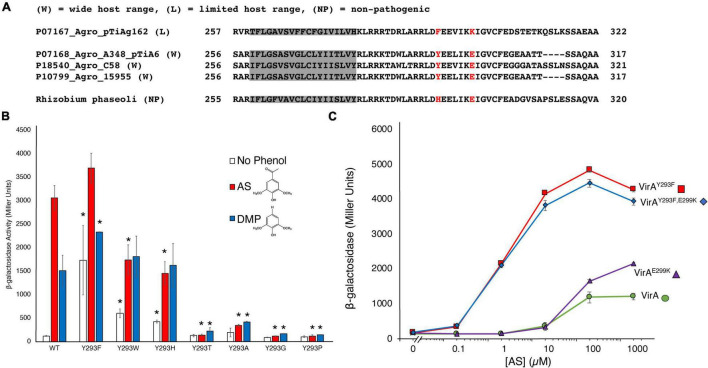
An aromatic residue in the SIN is critical for phenol perception. **(A)** Homology of the SIN regions from AlphaFold models and the non-pathogenic strain *Rhizobium phaseoli* are shown. A phenylalanine at the usual position for the tyrosine is found in the limited-host-range *A. tumefaciens* strain, while a histidine at this position is found for the *R. phaseoli* strain. **(B)** Mutations to Y293 were created using SLIM in pJZ6 plasmids, transformed with pRG106 into *A. tumefaciens* strain A136, and tested for β-galactosidase activity with 300 μM AS (red bars), 300 μM DMP (blue bars), or the absence of phenol (white bars). Error bars represent the standard deviation of three isolates. **(C)** β-galactosidase activity across varying AS concentration (μM) for four VirA constructs: VirA*^Y293F^* (red), VirA*^Y293F/E299K^* (blue), VirA*^E299K^* (purple), or VirA*^wt^* (green). All samples were supplemented with 1% (14 mM) glucose. Error bars represent the standard deviation of three isolates and the asterisks (*) represents a significant difference (*P* value < 0.05) when compared with the corresponding wild type value.

Different *virA* alleles from unique substrains have been implicated in determining the host-range of the bacterium, though these have mostly focused on the differences in promoter (Leroux et al., 1987; Turk et al., 1993). Of the four VirA proteins characterized by AlphaFold, three represent receptors from strains with a wide-host-range (UniProt P10799, P18540, P07168), with a tyrosine at position 293, and the fourth (UniProt P07167) has a limited-host-range (Figure 3A), with a phenylalanine at this position. Analysis of the AlphaFold prediction of the Linker regions of these VirA molecules show that their modeled structures are similar, though with slight structural differences (see Supplementary Figure 2). A recent study sequencing various bacteria from the rhizobial strains found that a non-pathogenic *Rhizobium phaseoli* strain also has a *virA* gene (Martinez et al., 1988; Wekesa et al., 2021). This VirA sequence has a histidine where the tyrosine and phenylalanine have been found in *A. tumefaciens* strains (Figure 3A). To our knowledge, this is the only other *virA* gene with an alternative residue beside tyrosine at this position in the SIN.

Overall, much of the response of VirA to signaling molecules has been well-characterized, especially with respect to large VirA truncations (Lin et al., 2014). In addition, single amino acid mutations in VirA have been identified using mutagenesis screens selecting for phenol specificity (Fang et al., 2015). In these screens, mutations to amino acid 293 were discovered to have critical effects to VirA signal response. Due to the dramatic effect of the Y293F mutation in creating an OR gate with heightened activity in response to phenol, we hypothesized that both aromaticity and hydrogen-bond interaction at this residue are important for phenol reception. We created several mutations at amino acid 293 to determine the effect of aromaticity and/or hydrogen-bond interaction on phenol reception (Figure 3B). Indeed, mutations that did not retain aromaticity at the 293 residue – VirA*^Y^*293*^A^*, VirA*^Y^*293*^P^*, VirA*^Y^*293*^T^*, and VirA*^Y^*293*^G^* – had dramatically lower response to AS and DMP. It is notable that these mutations also lack hydrogen-bond interaction capability, with the exception of threonine. Nonetheless, threonine could be too small to make bonding contact between the helices or allow VirA to maintain the structure required for strong phenol reception. Although we do not present structural data here to show that each of these mutants are folded and localized correctly, VirA*^Y^*293*^P^* and VirA*^Y^*293*^T^* were previously discovered in a specificity screen that revealed activity at high levels of DMP, indicating that these mutants may be functional (Fang et al., 2015).

In contrast to the mutants that remove aromaticity at position 293, VirA*^Y^*293*^W^* and VirA*^Y^*293*^H^* had a significant response to phenols (Figure 3B). These mutants, however, did not appear to have the OR/AND gating shift as seen in the VirA*^wt^* to VirA*^Y^*293*^F^* mutations. The VirA*^Y^*293*^W^* and VirA*^Y^*293*^H^* variants appear to have an “intermediate gating” phenotype and a loss of phenol specificity. Although they still retain aromaticity, the presence of a basic amino acid (histidine) in a hydrophobic pocket or presence of a sterically bulky tryptophan could have a destabilizing effect on the arrangement of the two helices, and consequently the Linker region as a whole. The VirA*^Y^*293*^W^* and VirA*^Y^*293*^H^* mutations have a response that neither represents what has been characterized as OR gating *via* VirA*^wt^*, requiring phenol for *vir* gene production, nor what has been characterized as AND gating *via* VirA*^Y^*293*^F^*, which has a strong response in the absence of phenols. This effect was confirmed in experiments with VirA*^Y^*293*^W^* and VirA*^Y^*293*^H^* where a range of AS concentration was used (Supplementary Figure 3). Additionally, while both VirA*^wt^* and VirA*^Y^*293*^F^* show a preference for AS over DMP, this effect is lost with the VirA*^Y^*293*^W^* and VirA*^Y^*293*^H^*. As the only other reported mutation to this region is found in a non-pathogenic strain (see Figure 3A), these experiments reveal the essential nature of the SIN, and the aromatic residue that resides in it, for phenol specificity and overall response.

Analysis of the differences between VirA molecules in wide-host-range and limited-host-range *A. tumefaciens* revealed differences beyond the tyrosine to phenylalanine at position 293 (Fang et al., 2015). Specifically, wide-host-range strains have an acidic residue at position 299, while there is a basic residue at this region in the limited-host-range strains (see Figure 3A). To test the possibility that this residue plays a significant, perhaps compensatory, role in phenol perception, we created VirA*^wt/E^*299*^K^* and VirA*^Y^*293*F/E*299*^K^* mutant strains. The VirA*^Y^*293*F/E*299*^K^* mutation begins to create a similar region in VirA as limited-host-range strain VirA. Despite these dramatic changes to the charged residues in this region of the protein, we did not observe much difference in the effect of AS response (Figure 3C). The lack of a difference here supports the AlphaFold model, as position 299 appears to be on the opposite face of 293 and is not interacting with helix α4 (see Figure 1C). For the limited-host-range protein, however, K300, the equivalent position as E299 in the wide-host-range protein, might be in a more crucial binding pocket, as predicted by the AlphaFold model of this protein (see Figure 1D).

### ASBr Inhibition and pH Affect OR/AND Gating Similarly

To further analyze the nature of how mutations to amino acid 293 affect VirA function, we synthesized ASBr, which has been shown to inhibit the VirA/VirG system (Lee et al., 1992). After chemical bromination to create ASBr (see section “Materials and Methods”), we added increasing amounts of ASBr in the presence of AS to observe whether inhibition could be observed in both the AND (VirA*^wt^*) or OR (VirA*^Y^*293*^F^*) gating strains (Supplementary Figure 4A). DIMBOA, a metabolite of maize that inhibits VirA in addition to ASBr, was previously shown to be ineffective at inhibiting VirA*^Y^*293*^F^* (Fang et al., 2015), but we found that a concentration of 100 αM ASBr can inhibit VirA*^Y^*293*^F^* phenol response. ASBr at a concentration greater than 100 μM, however, decreased *A. tumefaciens* growth so that interpretations of inhibition were compromised (Supplementary Figure 5A). The additional aromatic mutants VirA*^Y^*293*^H^* and VirA*^Y^*293*^W^* showed a similar response to ASBr (Supplementary Figure 5B). These data support the hypothesis that DIMBOA inhibition is mechanistically distinct from ASBr inhibition, which is presumed to be a competitive inhibitor of VirA (Hess et al., 1991).

The AlphaFold model of VirA hypothesizes that there is a connection between its periplasmic region and Linker region through a single helix, α1. If the AlphaFold model is correct, α1 could transmit a pH-dependent sugar signal from the periplasmic space to the Linker region (McCullen and Binns, 2006). While we use the AND/OR nomenclature here, we recognize that there is a spectrum of response. For instance, while the AND gated VirA*^wt^* does have a very low response to AS in the absence of sugar, OR gated VirA*^Y^*293*^F^* has a more sensitive and higher maximal response to AS without sugar (Fang et al., 2015). To further probe the model of signal integration, we tested whether logic gating was affected by a pH change. Low pH (5.5) is known to be important for VirA response, and we observed this similar phenotype with both the AND (VirA*^wt^*) and OR (VirA*^Y^*293*^F^*) gated strains in the presence or absence of 1% glucose (1% glycerol must be added as a carbon source in the absence of glucose) (Supplementary Figure 4B). Though a pH of 7.5 lowered its overall response, the OR gated strain maintained a similar relative increase in signal response regardless of the availability of the inducing glucose.

### Identification of a Potential Phenol-Binding Pocket

**FIGURE 4:**
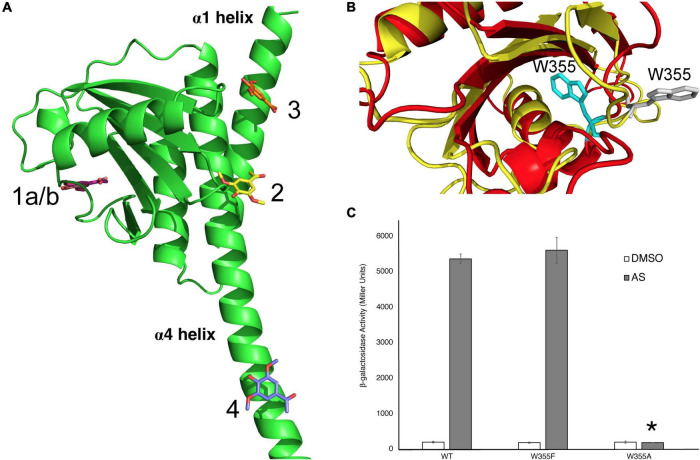
Identification of W355 as a critical amino acid for phenol interaction. **(A)** The modeling program GOLD was used to predict where the phenol AS could bind the Linker region. Interactions with binding pockets near W355 (1), Q427 (2), R444 (3), and R454 (4) are shown. **(B)** Two models of the VirA Linker, from AlphaFold in red and Phyre2 in yellow, show different predictions for the orientation of the W355 residue. For the AlphaFold model, W355 is shown in gray and for the Phyre2 model, it is shown in cyan. **(C)** β-galactosidase activity of VirA*^wt^*, VirA*^W355F^*, and VirA*^W355A^* alleles in pJZ6 plasmids with pRG106 in *A. tumefaciens* strain A136 in the absence (□) or presence (■) of AS, supplemented with 1% (14 mM) glucose. Error bars represent the standard deviation of three isolates and the asterisk (*) represents a significant difference (*P* value < 0.005) when compared with the corresponding wild type value.

The AlphaFold Model of the Linker region was used to predict where AS could bind the Linker domain using the GOLD docking program, and four potential binding sites were identified (Figure 4A). The binding sites include separate binding cavities with the following amino acids in close proximity to the predicted AS binding site: W355 (1), Q427 (2), R444 (3), and R454 (4). These pockets contain numerous potential interactions between AS and neighboring residues (Supplementary Figure 6). We also identified similar putative binding sites using the Linker model generated by Phyre2, as reported previously (Lin et al., 2014). However, these two models, AlphaFold and Phyre2, show contradicting predictions for the conserved W355 orientation in the Linker domain (Figure 4B). While Phyre2 shows that this tryptophan may be in an accessible orientation for AS binding, the AlphaFold model predicts that this tryptophan is less accessible. To test the importance of this residue in VirA phenol response, we created VirA*^W^*355*^F^* and VirA*^W^*355*^A^* mutations. Our data show that W355 does appear to be critical for VirA phenol response, as the VirA*^W^*355*^A^* mutant is unable to induce in the presence of AS (Figure 4C). However, the VirA*^W^*355*^F^* recovers phenol response, potentially indicating that this residue provides structural support for the Linker. Further structural studies will be necessary to determine if this is a true phenol binding pocket and whether the VirA*^W^*355*^A^* mutant causes a significant deviation from proper protein folding. Additionally, Q427, which is located on the helix α4 in the model, is predicted to make both hydrogen-bond interactions with Arg289 located on helix α1, and the N-terminus residue Tyr5 (Supplementary Figure 7). Mutations that disrupt this potential interaction, such as our insertions in Figure 2 and 293 mutations in Figure 3, may be physically preventing phenol interactions in this region, and further experiments should investigate these possibilities.

## Discussion

**FIGURE 5:**
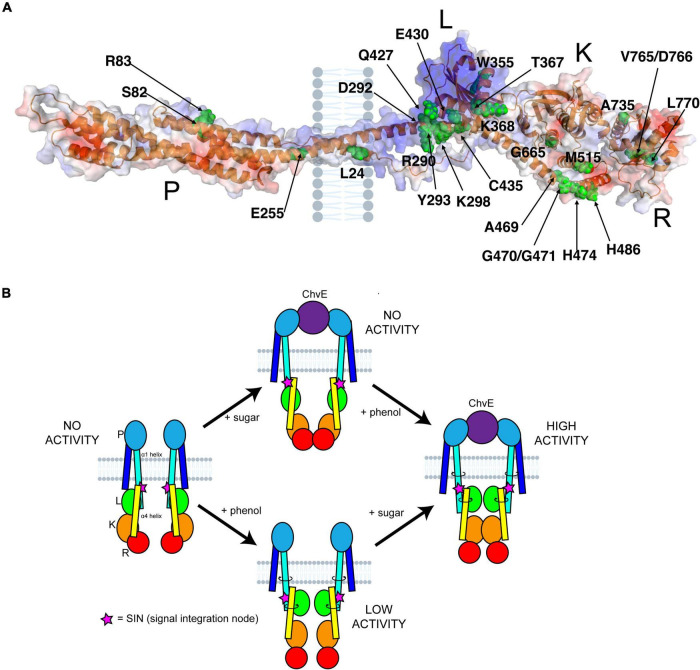
VirA structure and signal coordination. **(A)** Previously reported residues in VirA were mapped onto the AlphaFold prediction (Pan et al., 1993; Banta et al., 1994; McLean et al., 1994; Nair et al., 2011; Lin et al., 2014; Fang et al., 2015). These residues include sugar perception in the Periplasmic (P) region, phenol perception in the Linker (L) region, kinase activity in the Kinase (K) region, and regulation in the Receiver (R) region. The putative position of the inner membrane is shown. **(B)** A comprehensive model of VirA activity can be presented. Here, the options of phenol or sugar input are shown, with a direct link from periplasm to cytosol through the α1 helix, that can twist and ratchet the α4 helix upon an interaction with sugar-bound ChvE and/or phenol. The signal integration node (SIN) (pink stars), of critical importance to VirA signal perception, is shown as well.

Unlike most experimentally characterized two-component systems, the VirA-VirG system coordinates the reception of multiple signals (McCullen and Binns, 2006; Perry et al., 2011; Capra and Laub, 2012). Through AlphaFold modeling and the data generated here, we can contextualize previous research and begin to uncover this molecular mechanism to answer decades-old questions about how VirA coordinates these signals. Using our AlphaFold model and the important VirA residues as identified in the literature, we generated an image that orients these residues to the full protein (Figure 5A). We have included the putative position of the inner membrane as well, which fits previous VirA membrane-spanning predictions (Nair et al., 2011). Here, we can specifically observe a cluster of residues in the Linker region, especially at the interface between two α-helices, α1 and α4, that appear to coordinate and propagate signal for the VirA model.

The AlphaFold predictive model can be used to refine the model for how VirA might coordinate the two physical signals necessary for full induction of the *vir* genes. Upon sugar binding, ChvE interacts with the VirA dimer in the periplasm, and this new model shows how this signal might be transmitted directly through the membrane-spanning 70 amino acid helix α1 (see Figures 1A, 5B). Toward the C-terminal end of this helix, the SIN provides interactions that are critical for determining phenol specificity. In particular, we have shown that the nature of amino acid 293 is of paramount importance to both interact with phenols and allow VirA to specify between the phenols AS and DMP. As we have presented through insertion mutations and amino acid substitutions, structural integrity at the SIN impacts phenol response and further experiments will be necessary to show that the mutants presented here are able to form functional dimers in the inner membrane. While these models from AlphaFold provide a framework for understanding these structures, structural experiments must be performed to test these models. Though expressing and purifying membrane bound proteins can be challenging, recent work with cryoEM has been able to characterize similarly sized membrane-bound structures, and the dimer nature of VirA may facilitate structural work along these lines (Nogales and Scheres, 2015).

The new structural modeling has allowed us to synthesize several previous observations into one scheme. In Figure 5B, we synthesize the mechanistic predictions for VirA activity that have been produced in this work and in the literature (Hess et al., 1991; Wang et al., 2002; McCullen and Binns, 2006; Gao and Lynn, 2007; Lin et al., 2014). Previous models highlighted the importance of the TM2 region to provide the ratcheting necessary to transmit the ChvE-sugar interaction that occurs in the periplasmic space, but these models separated the Periplasmic region from the Linker region (Lee et al., 1992; Wang et al., 2002; Gao and Lynn, 2007; Nair et al., 2011). With a single helix connecting the periplasmic region to the Linker region through the inner membrane of the bacterium, as predicted by AlphaFold, there is a direct mechanism for ratcheting once the Periplasmic region interacts with a sugar-bound ChvE. This ratchet could strengthen the interaction of the Linker dimer, providing an even more robust response in the presence of both phenol and sugar, and then allow α1 (aa 233–302) to ratchet α4 (aa 423–492) and orient the Kinase region of VirA. Our new model of VirA signal coordination now includes the previous ratcheting hypothesis (Gao and Lynn, 2007; Lin et al., 2014), but provides a direct structural implication of sugar-interaction in the periplasm, through phenol binding in the Linker, to the alignment of the histidines involved in auto-phosphorylation and signal propagation.

The Linker region putatively binds phenol, as several experiments and observations have led to this hypothesis. First, though VirA without the periplasmic and linker regions, VirA(KR), can constitutively activate virulence response, there is no effect when phenol is added (Lin et al., 2014). Indeed, insertions to the proposed α-helix that lead into the kinase domain propagate a ratcheting signal that activates this domain (Gao and Lynn, 2007). In contrast to VirA(KR), a mutation that is lacking the periplasmic region alone, VirA(LKR), cannot respond to sugar through the periplasmic region but can respond to specific phenolic signals, providing supporting evidence for phenol binding in this region. Multiple structural prediction programs have modeled the Linker region as a GAF domain, including the AlphaFold program, and direct phenol interaction still seems the most likely explanation for VirA phenol response. The GOLD modeling software used here allows us to make predictions of AS interaction with the Linker region using this AlphaFold model. Through the creation of amino acid substitutions at this region, we have shown that one of these binding pockets, W355, appears to be critical for phenol response, but we have yet to determine whether this is a direct binding site of AS or leads to structural instability that prevents interaction elsewhere in the protein. Further perturbations to this region of VirA will elucidate how the VirA*^W^*355*^A^* leads to phenol insensitivity. Additionally, the inhibitors ASBr and DIMBOA seem to have different effects on VirA phenol-sensitive mutants, indicating that there may be multiple regions of VirA that contribute to its ability to respond to phenol.

The AlphaFold predictive model also provides a reference point to further examine and test the role of the regulatory regions of VirA. While the Receiver domain has traditionally been thought of as a negative regulator of VirA activity, recent evidence suggests that this region has a stimulatory effect dependent on how the downstream factor VirG is expressed in the experiment (Wise et al., 2010; Wise and Binns, 2016). In addition, ChvE-sugar may have a phenol-independent inducing effect that can only be revealed in mutants to the regulatory regions of VirA (Wise and Binns, 2016). Our mutations to residue 293 here show a phenol-independent response phenotype as well, indicating that this region is important for signal regulation. Through our reporting of a predictive model of VirA structure, we can begin to probe the mechanism by which the Receiver domain might act on the Linker/Kinase regions through physical interactions, potentially through revealing or occluding phenol-binding, phosphorylation, or VirG-binding sites.

Finally, primary structure sequence differences of VirA might contribute directly to its host range, and here we have shown that the three AlphaFold models of wide-host-range VirA are somewhat different from the limited-host-range VirA (see Supplementary Figure 2A). This, coupled with observations detailing the importance of the *virA* promoter, provides a hypothesis for how the VirA signaling molecule might be responsible for the host range of *A. tumefaciens* (Turk et al., 1993; Lee et al., 1998). While the majority of experiments regarding VirA constructs have focused on using sequence information from wide-host-range isolates, further analysis and testing of the structural predictions using VirA isolated from limited-host-range subspecies will be necessary for determining how structural changes to signal reception might lead to *A. tumefaciens* host response.

The AlphaFold model and data presented here both confirm previous observations and contribute to a more comprehensive model of VirA function. *A. tumefaciens* must be able to respond to specific signals and coordinate its pathogenic response in the presence of a multitude of competing molecules. This model provides a new structural framework for how VirA responds to these signals and the success of using a predictive modeling system could lead to further understanding of signal reception and propagation in similar two-component systems across multiple organisms.

## Data Availability Statement

The raw data supporting the conclusions of this article will be made available by the authors, without undue reservation.

## Author Contributions

BP designed the experiments and wrote the manuscript. AKD and MKS performed and described the PyMoL experiments and molecular modeling. AS, EAPP, JG, SD, KG, NRV, MK, AP, AK, and RD contributed to the construction of plasmids used and performed the beta-gal experiments. BP, AKD, MKS, AS, EAPP, JG, SD, KG, NRV, and MK contributed to the writing of the manuscript. All authors contributed to the article and approved the submitted version.

## Conflict of Interest

The authors declare that the research was conducted in the absence of any commercial or financial relationships that could be construed as a potential conflict of interest.

## Publisher’s Note

All claims expressed in this article are solely those of the authors and do not necessarily represent those of their affiliated organizations, or those of the publisher, the editors and the reviewers. Any product that may be evaluated in this article, or claim that may be made by its manufacturer, is not guaranteed or endorsed by the publisher.
