# Supplementary material for: Mechanistic Analysis of the VirA Sensor Kinase in Agrobacterium tumefaciens Using Structural Models
Source: Front Microbiol. 2022 May 16;13:898785. doi: 10.3389/fmicb.2022.898785 (PMC9149312; doi:10.3389/fmicb.2022.898785)

**Supplementary Table 1: Primers used in this study**

| Primer | Characteristic | Sequence (5’🡪3’) (Tailed primer, *mutation*, **insertion**, restriction site, stop) |
| --- | --- | --- |
|  |  |  |
| DP164 | VirA_rev | GCGGTACCTCACTACGTCTTGATTTTGGTTAG |
| DP204 | VirA_for | GAGGATCCATGAACGGAAGATATTCACCGACG |
| DP270 | W355A_for | AAA CCT GTG *GCG* GAC GAC AGC GTG CTA CGC GAA ATA GTC TCT CGT ACC |
| DP271 | W355A_rev | CAC GCT GTC GTC *CGC* CAC AGG TTT TGG GTG TTT CGC ACC GAA TGT TTC |
| DP276 | aa354-357_for | CTA CGC GAA ATA GTC TCT CGT ACC |
| DP277 | aa354-357_rev | TGG GTG TTT CGC ACC GAA TGT TTC |
| DP278 | Y293A_for | GAT *GCA* GAA GAG CTA ATC AAA GAG ATC GGA GTA TGT TTT GAA GGT GAG |
| DP279 | Y293A_rev | GAT CTC TTT GAT TAG CTC TTC *TGC* ATC TAA ACG CCG CGC TAA CCA ATC |
| DP280 | Y293G_for | GAT *GGA* GAA GAG CTA ATC AAA GAG ATC GGA GTA TGT TTT GAA GGT GAG |
| DP281 | Y293G_rev | GAT CTC TTT GAT TAG CTC TTC *TCC* ATC TAA ACG CCG CGC TAA CCA ATC |
| DP286 | Insertion of 3 Ala after 280_for | CTA **GCA GCA GCA** CGC AAA AAA ACC GAT TGG TTA GCG CGG CGT TTA GAT TAC GAA GAG |
| DP287 | Insertion of 3 Ala after 280_for | CGG CGT TTA GAT TAC GAA GAG |
| DP288 | Insertion of 3 Ala after 280_rev | CGC TAA CCA ATC GGT TTT TTT GCG **TGC TGC TGC** TAG CCT ATA GAC TAA GGT GAT GAT |
| DP289 | Insertion of 3 Ala after 280_rev | CCT ATA GAC TAA GGT GAT GAT |
| DP292 | Y293P_for | GAT *CCG* GAA GAG CTA ATC AAA GAG ATC GGA GTA TGT TTT GAA GGT GAG |
| DP293 | Y293P_rev | GAT CTC TTT GAT TAG CTC TTC *CGG* ATC TAA ACG CCG CGC TAA CCA ATC |
| DP294 | Insertion of 3 Ala after 285_for | GAT **GCA GCA GCA** TGG TTA GCG CGG CGT TTA GAT TAC GAA GAG CTA ATC AAA GAG |
| DP295 | Insertion of 3 Ala after 285_for | GAA GAG CTA ATC AAA GAG ATC |
| DP296 | Insertion of 3 Ala after 285_rev | ATC TAA ACG CCG CGC TAA CCA **TGC TGC TGC** ATC GGT TTT TTT GCG TAG CCT ATA |
| DP297 | Insertion of 3 Ala after 285_rev | GGT TTT TTT GCG TAG CCT ATA |
| DP298 | Insertion of 3 Ala after 293_for | TAC **GCA GCA GCA** GAA GAG CTA ATC AAA GAG ATC GGA GTA TGT |
| DP299 | Insertion of 3 Ala after 293_for | ATC AAA GAG ATC GGA GTA TGT |
| DP300 | Insertion of 3 Ala after 293_rev | GAT CTC TTT GAT TAG CTC TTC **TGC TGC TGC** GTA ATC TAA ACG CCG CGC TAA |
| DP301 | Insertion of 3 Ala after 293_rev | ATC TAA ACG CCG CGC TAA |
| DP318 | W355F_for | AAA CCT GTG *TTC* GAC GAC AGC GTG CTA CGC GAA ATA GTC TCT CGT ACC |
| DP319 | W355F_rev | CAC GCT GTC GTC *GAA* CAC AGG TTT TGG GTG TTT CGC ACC GAA TGT TTC |

**Figure S1. Complete CLUSTAL sequence alignment of AlphaFold modeled and *R. phaseoli* VirA proteins.** The four AlphaFold VirA proteins (UniProt P07167, P07168, P10799, and P18540) and the *Rhizobium phaseoli* VirA protein were aligned using CLUSTAL O(1.2.4).


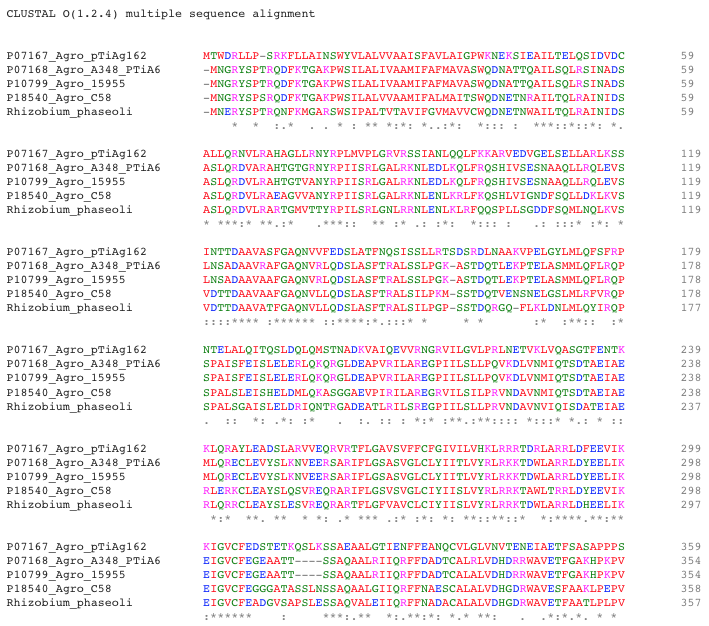


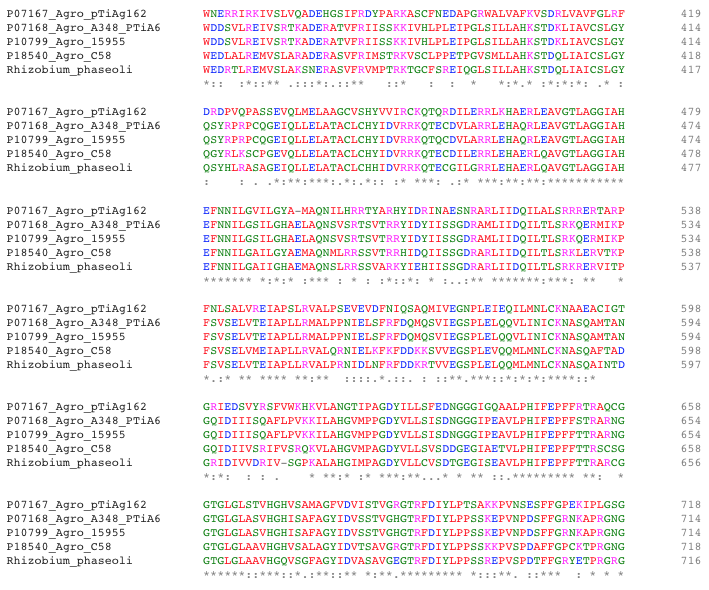


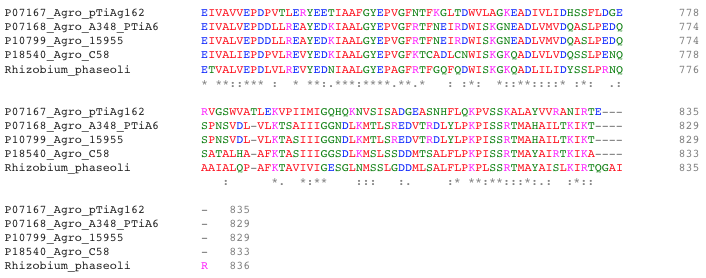

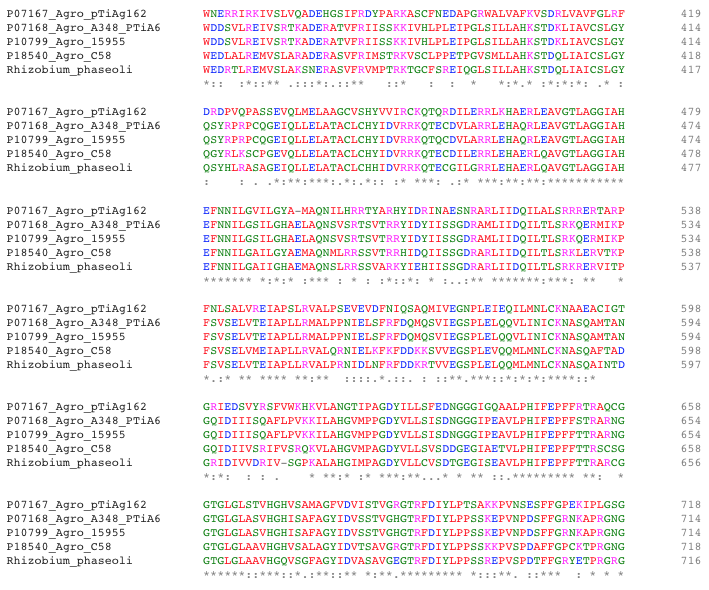


**Figure S2. Comparison of the full protein and Linker models from AlphaFold.** (A) The online structure prediction program, Alpha Fold (<https://alphafold.ebi.ac.uk/>) was used to predict the structure of VirA from 4 different versions of full-length VirA (P10799 in red, P18540 in yellow, P07168 in green, and P07167 in blue). The .pdb files of the suggested models were downloaded and aligned in the Pymol software to visualize the relative positioning of the different models to each other. The fold looks similar for the four predictions. (B) The Linker region of the best model P10799 was used to align with the other Linker models with a similar color scheme as in (A), and showed relatively similar structural fold, as indicated by the RMSD reported values. (C) For clarity, P07168 (green) and P07167 (blue), are shown without the other two structures. (D) The Linker regions of P07168 (green) and P07167 (blue) alone, which do show a high degree of similarity when isolated from the rest of the protein. The RMSD of these two structures is 1.562.

**
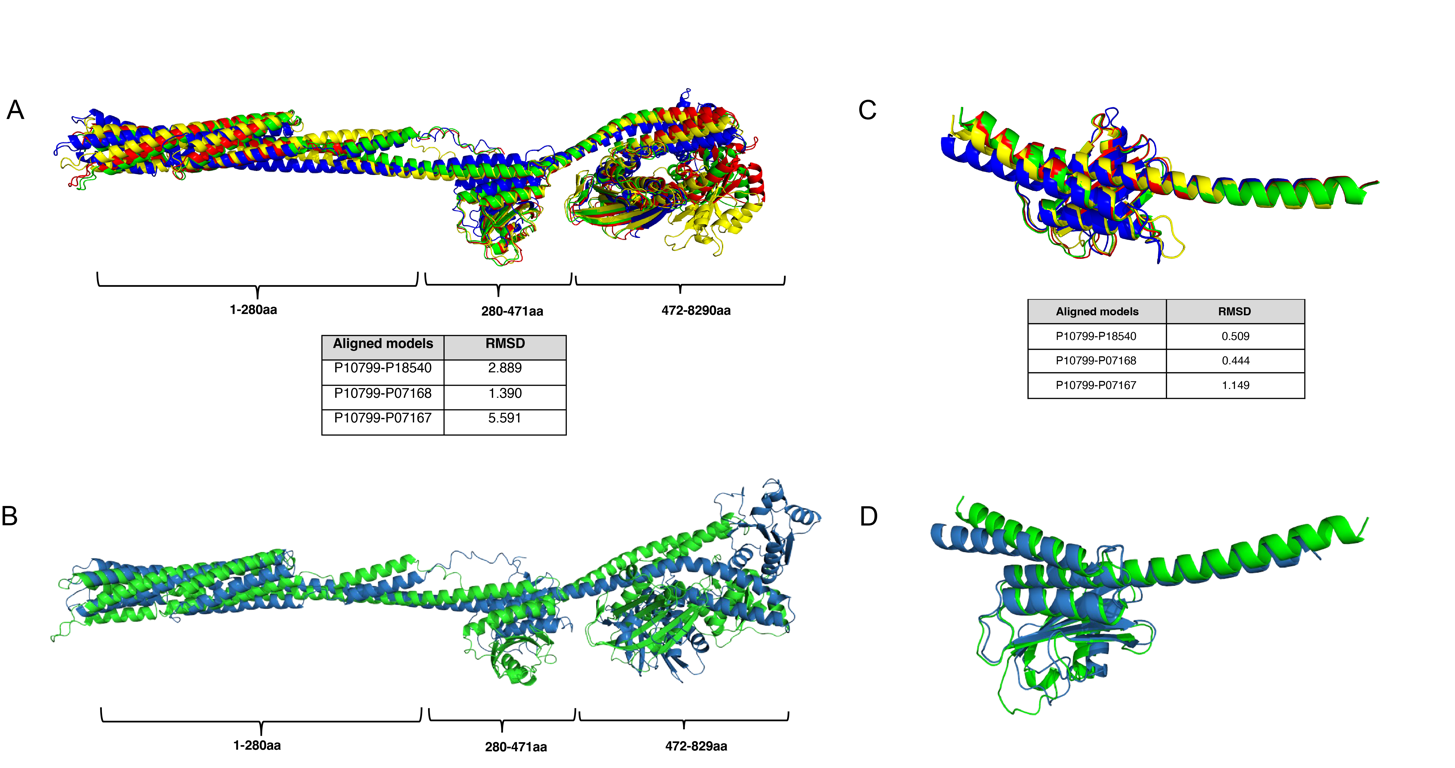
**

**Figure S3. Mutations to Y293 alter VirA phenol perception.** Compiled data of β-galactosidase activity for strains of *A. tumefaciens* containing VirA^wt^ (purple triangles), VirA^Y293F^ (blue diamonds), VirA^Y293H^ (green circles), or VirA^Y293W^ (red squares). Varying amount of acetosyringone were added and all samples contained 1% glucose, an inducing sugar. Error bars represent the standard deviation of three isolates.


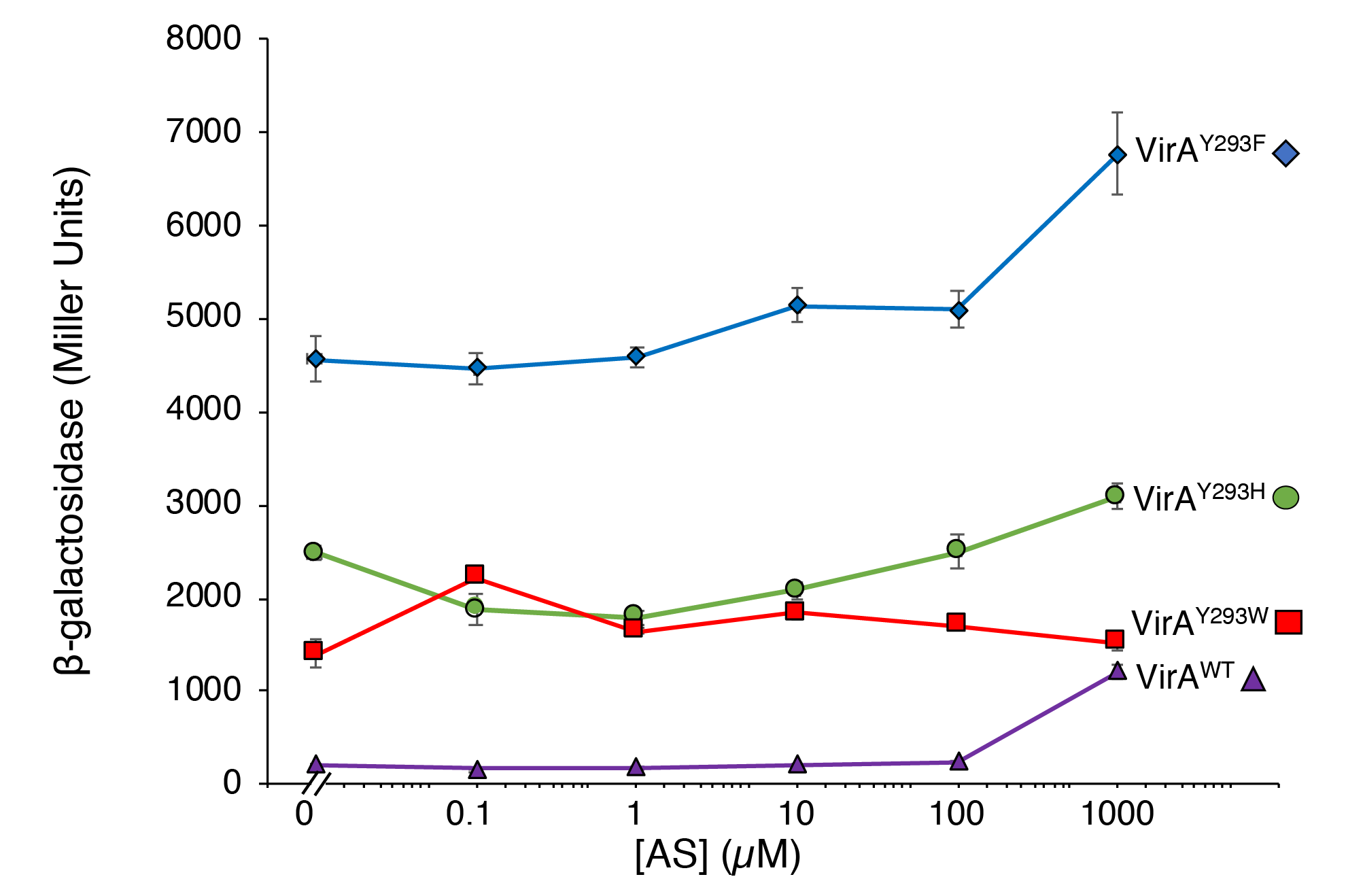


**Figure S4. AND/OR gating is both similarly inhibited by ASBr and affected by pH change.** (A) VirA^wt^ (AND gate) and VirA^Y293F^ (OR gate) induction activity was examined in the presence of varying amounts of the inhibitor ASBr. All *A.tumefaciens* cultures were supplemented with 300 µM AS and 1% (14 mM) glucose. Error bars represent the standard deviation of three isolates. (B) β-galactosidase activity was determined for the strains VirA^wt^ (AND gate) and VirA^Y293F^ (OR gate) in varying pH (5.5 and 7.5) as shown. Strains were grown in 1% glycerol (🞏) or 1% glucose (◼), and supplemented with 300 µM AS. Error bars represent the standard deviation of three isolates.

**
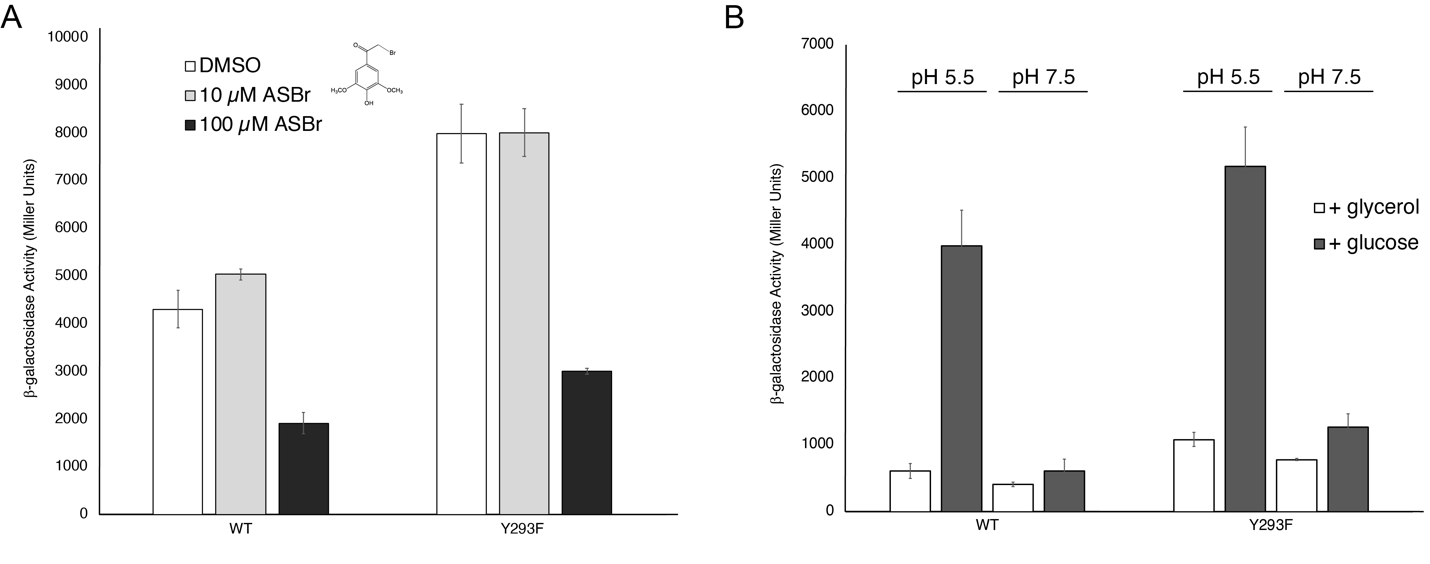
**

**Figure S5. VirA mutant reaction to ASBr.** (A) Growth of VirA^WT^ and VirA^Y293F^ in the presence of increasing amounts of ASBr. All strains were grown in the presence of 1% glucose and 300 µM AS. (B) Activity of VirA^WT^, VirA^Y293F^, VirA^Y293W^, and VirA^Y293H^ in the presence of 0, 10µM, and 100 µM ASBr. Two separate experiments were combined and therefore activity is expressed as a fraction of the maximal β-galactosidase activity. Error bars represent the standard deviation of three isolates.


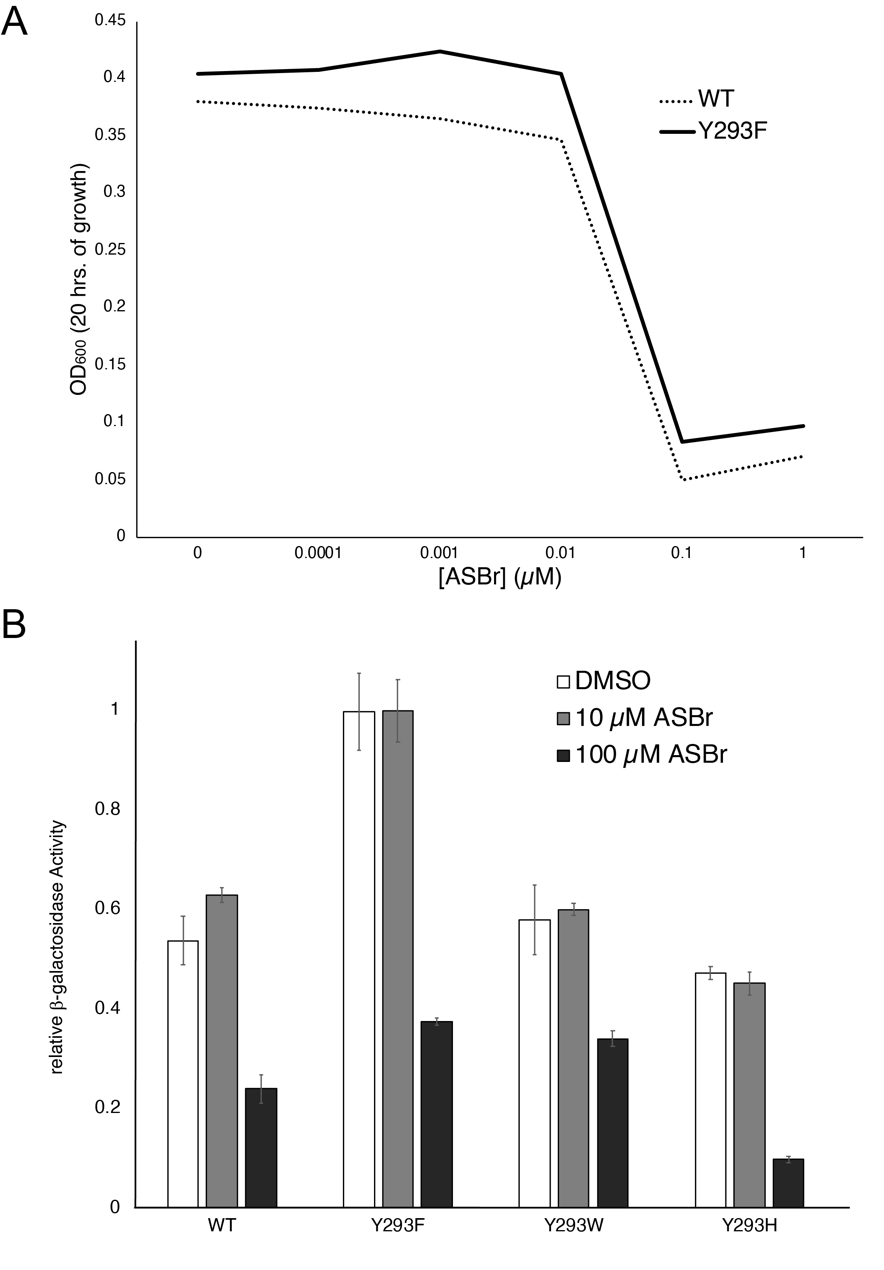


**Figure S6. Putative AS binding sites in the Linker region of VirA.** Each binding site was determined using the GOLD modeling program. (A) Docking of AS in the W355 binding pocket (position 1a in Fig. 5A) with the W355 pointing outward (see Fig. 5B), as in the Phyre2 model. (B) Docking of AS in the W355 binding pocket (position 1b in Fig. 5A) with the W355 pointing inward (see Fig. 5B), as in the AlphaFold model. (C) Docking of AS in the R444 cavity (position 2 in Fig. 5A). (D) Docking of AS in the Q427 cavity (position 3 in Fig. 5A). (E) Docking of AS in the R454 cavity (position 4 in Fig. 5A).


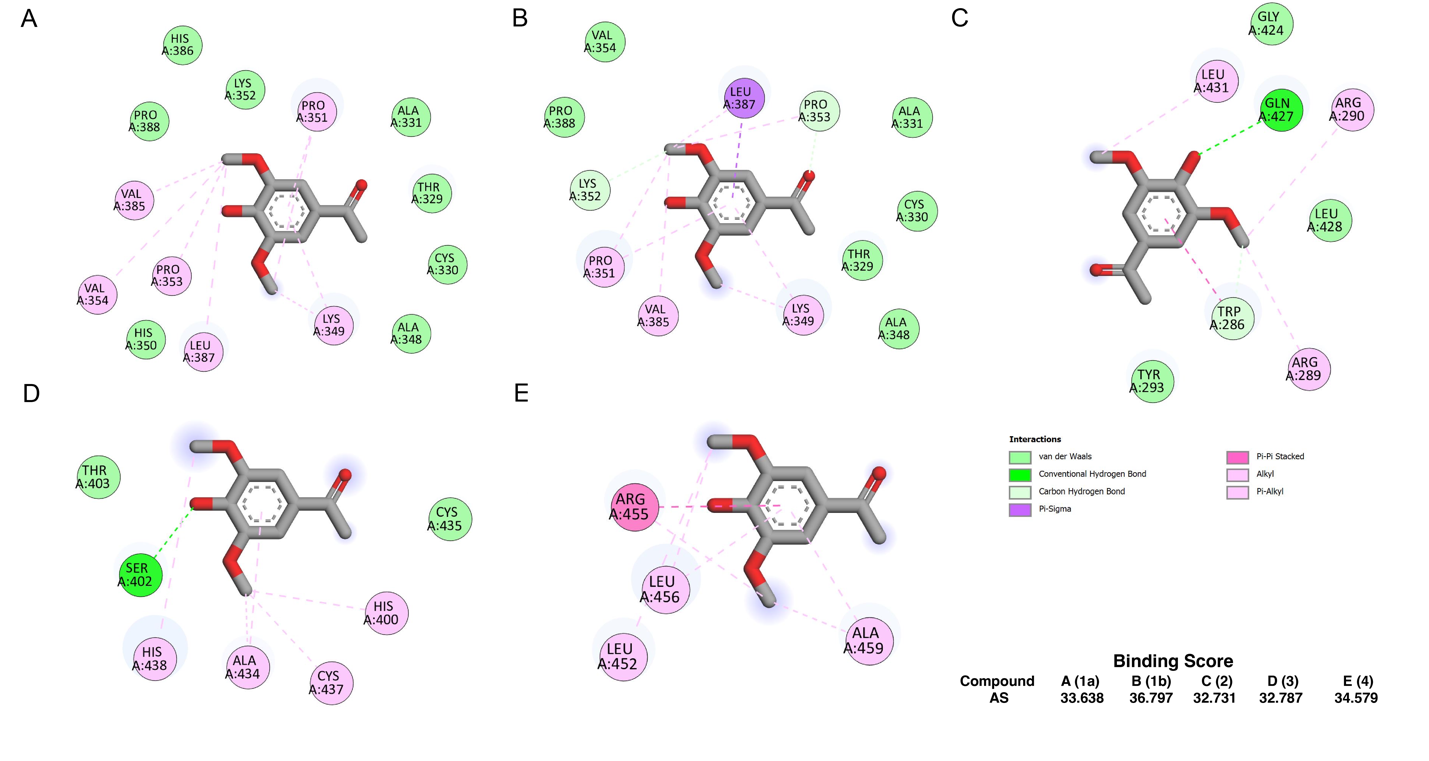


**Figure S7. Modeled interaction between VirA regions** (A) The AlphaFold modeled structure of VirA (UniProt P07168) predicts a potential interaction between Q427, R289, and Y5. (B) A closer view of the interaction between these three amino acids, which may allow for further regulation of VirA signal perception.


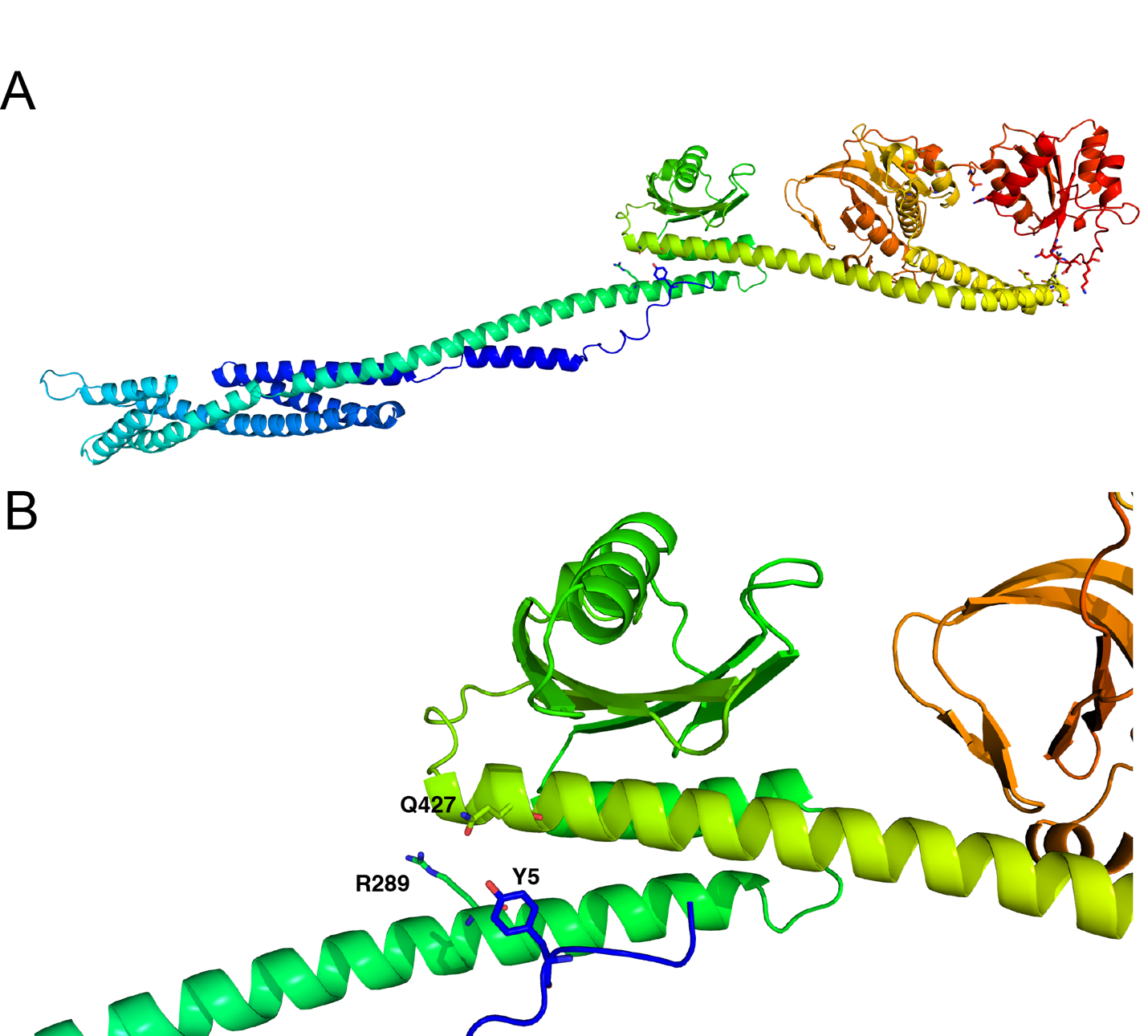

Supplement: Supplementary file 1 [file Data_Sheet_1.docx]
